# Alzheimer Cells on Their Way to Derailment Show Selective Changes in Protein Quality Control Network

**DOI:** 10.3389/fmolb.2020.00214

**Published:** 2020-11-20

**Authors:** Margreet B. Koopman, Stefan G. D. Rüdiger

**Affiliations:** ^1^Cellular Protein Chemistry, Bijvoet Center for Biomolecular Research, Utrecht University, Utrecht, Netherlands; ^2^Science for Life, Utrecht University, Utrecht, Netherlands

**Keywords:** Alzheimer’s disease, chaperones, proteostasis, autophagy, stress response, proteomics

## Abstract

Alzheimer’s Disease is driven by protein aggregation and is characterized by accumulation of Tau protein into neurofibrillary tangles. In healthy neurons the cellular protein quality control is successfully in charge of protein folding, which raises the question to which extent this control is disturbed in disease. Here, we describe that brain cells in Alzheimer’s Disease show very specific derailment of the protein quality control network. We performed a meta-analysis on the Alzheimer’s Disease Proteome database, which provides a quantitative assessment of disease-related proteome changes in six brain regions in comparison to age-matched controls. We noted that levels of all paralogs of the conserved Hsp90 chaperone family are reduced, while most other chaperones – or their regulatory co-chaperones - do not change in disease. The notable exception is a select group consisting of the stress inducible HSP70, its nucleotide exchange factor BAG3 – which links the Hsp70 system to autophagy - and neuronal small heat shock proteins, which are upregulated in disease. They are all members of a cascade controlled in the stress response, channeling proteins towards a pathway of chaperone assisted selective autophagy. Together, our analysis reveals that in an Alzheimer’s brain, with exception of Hsp90, the players of the protein quality control are still present in full strength, even in brain regions most severely affected in disease. The specific upregulation of small heat shock proteins and HSP70:BAG3, ubiquitous in all brain areas analyzed, may represent a last, unsuccessful attempt to advert cell death.

## Introduction

Neurodegenerative diseases are a group of diseases characterized by progressive neuronal degeneration, of which Alzheimer’s Disease (AD) is the most prominent one. Symptoms of AD include severe memory loss and cognitive decline and are often accompanied by changes in personality (Bature et al., 2017). On molecular level, AD shows the accumulation of two distinct proteins; extracellular plaques of amyloid-β and intracellular formation of neurofibrillary tangles of Tau; a microtubule associated protein assisting in microtubule stability and regulating axonal transport (Drubin and Kirschner, 1986; Goedert et al., 2017). Under pathological conditions, Tau dissociates from the microtubules and aggregates into Tau fibrils, a process ultimately leading to cellular death. However, the exact underlying molecular mechanism of Tau aggregation is still unknown.

The protein quality control (PQC) system plays a crucial role in protein folding, prevention of aggregation and controlling protein degradation. Members of the two major ATP-dependent chaperone families, Hsp70 and Hsp90, are key players in PQC. Other conserved members of the metazoan PQC network are the small heat shock proteins and the Hsp60 chaperonins, which are associated with neurodegenerative diseases (Meriin and Sherman, 2005; Webster et al., 2019).

The Hsp70-Hsp90 folding cascade has a key role in PQC and is present in various cellular compartments. The ATP-dependent chaperone Hsp70 acts as an unfoldase in the early phase of the folding cascade and after repetitive cycles of binding and release transfers its substrate for further maturation to Hsp90 (Morán Luengo et al., 2019). Hsp90 is also an ATP-dependent chaperone and acts in the decision making of its substrate, directing it either along the folding or degradation pathway (Connell et al., 2001). Next to their active role in protein folding, the chaperones also play a role in protein aggregation or disaggregation processes. The Hsp70 system acts as a disaggregation machinery for several amyloidogenic proteins (Gao et al., 2015; Ferrari et al., 2018; Scior et al., 2018; Nachman et al., 2020). Both Hsp70 and Hsp90 are known to interact with Tau and have a role in normal Tau regulation but also in cell stress, protein aggregation and degradation, implying a role for both chaperones in AD (Dickey et al., 2007; Karagöz et al., 2014; Kundel et al., 2018; Ferrari et al., 2020; Weickert et al., 2020).

If folding fails, the PQC has different degradation pathways to deal with protein misfolding and aggregation. The ubiquitin proteasomal system (UPS) degrades approximately 80–90% of proteins which are mostly short-lived, denatured or damaged (Finley, 2009). Long-lived protein aggregates are sequestered for removal by the autophagic lysosomal system (Lilienbaum, 2013). The autophagy system comprises micro-autophagy, macro-autophagy and chaperone mediated autophagy, of which the latter two are linked to the Hsp70 system. One specific type of macro-autophagy – the chaperone assisted selective autophagy – selectively degrades ubiquitin-positive substrates. The substrates are targeted towards this pathway by a complex of Hsp70:BAG3, allowing the formation of an autophagosome, which will be degraded (Arndt et al., 2007). Interestingly, BAG3 increases clearance of PolyQ aggregates via the autophagy pathway (Carra et al., 2008) raising the question whether this could also play an important role for Tau aggregates in AD pathology.

As neurons can survive for decades despite the continuous presence of seeding-competent Tau protein, the PQC must be in good shape under normal conditions, in particular the Hsp70 and Hsp90 machines. This raises the question why after so many years Tau enters a fatal aggregation process, and to which extent derailment in one or more of the PQC pathways may take center stage in this. Multiple studies in cell lines or animal models have shown how individual components or pathways are associated with AD (Klaips et al., 2018; Schaler and Myeku, 2018; Hegde et al., 2019). Studies monitoring mRNA levels detected some adaptions of the quality control system in Alzheimer brains (Brehme et al., 2014; Fu et al., 2019). Several wide-scale proteomic studies have been performed to test whether networks or pathways have altered in aging or AD (Sultana et al., 2007; Donovan et al., 2012; Walther et al., 2015; Johnson et al., 2020). It is unclear, however, whether and to which extent the PQC capacity specifically is reduced in the human AD brain.

Recently, the Unwin group performed a wide-scale proteomics analysis on protein levels of nine AD brains in comparison to nine age-matched healthy controls (Xu et al., 2019). Their analysis revealed that an AD brain shows significant changes in specific signaling pathways, including the innate immune response and pathways involved in cell cycle regulation and apoptosis. They observe an association between the extend of affectedness of brain regions and protein level changes, implying a gradual change over the course of AD pathology. Notably, the study also revealed up- or down regulation of several individual PQC factors, such as an increase in heat-shock inducible HSP70, downregulation of the J-protein DNAJC6 and a strong increase for nucleotide-exchange factor BAG3 and small heat shock protein HSPB1. Chaperones, however, do not act as lone players, they cooperate as part of a large network. The network nature means that the PQC system could be derailed by upregulation of some factors while other members may be downregulated. Therefore, we now aim to reveal a comprehensive picture on alterations in levels of the PQC system by performing a meta-analysis on the extensive dataset provided by the Unwin group in the freely accessible Alzheimer’s Disease Proteome database (Xu et al., 2019).

We analyzed a plethora of proteins who all have distinct roles in the PQC and looked for changes in any of the PQC pathways. We noticed a decrease of all Hsp90 paralogs in the cytoplasm, mitochondria and endoplasmic reticulum (ER), as well as strong upregulation of the stress-regulated pathway preparing proteins for autophagy-mediated removal. These differences are indicative of cellular distress and point toward recruitment of multiple degradation pathways for the cell trying to remove protein aggregates.

## Results

### Rational of the Approach

**FIGURE 1:**
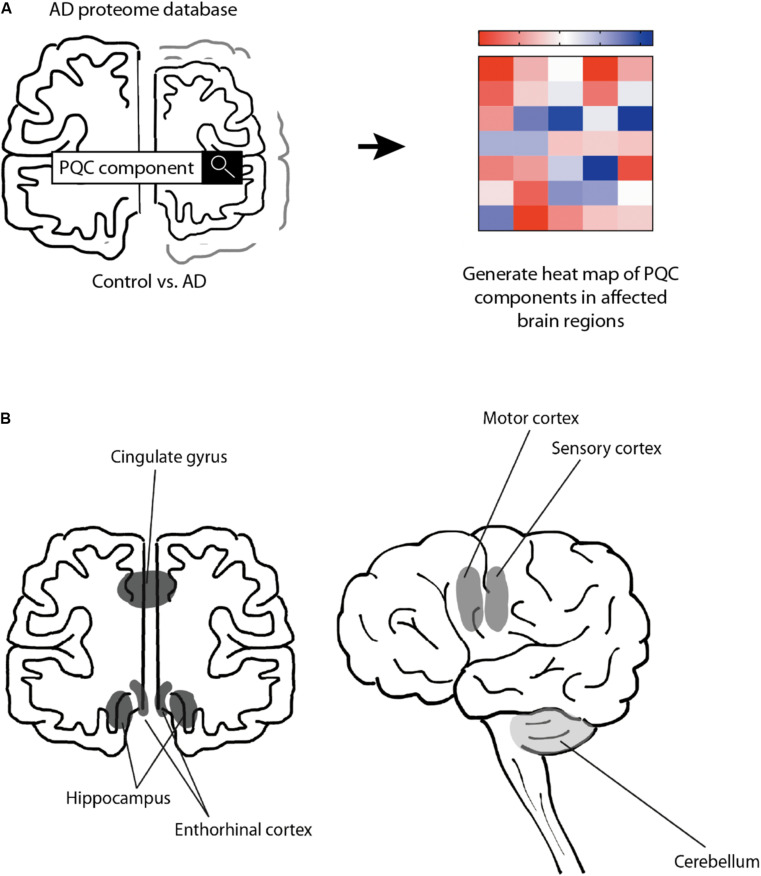
Schematic representation of approach. Schematic overview of the rational of our analysis. **(A)** Protein expression levels were obtained from the Alzheimer’s Disease Proteome Database and compared between 9 AD brains and 9 age-matched control brains (Xu et al., 2019). Heat-map was created to illustrate the protein level changes according to brain region. **(B)** Schematic representation of analyzed brain regions. Brain regions are colored along a gray gradient by affectedness, ranging from mostly unaffected (cerebellum, light gray) to mildly affected (sensory and motor cortex, medium gray), to severely affected (hippocampus, entorhinal cortex and cingulate gyrus, dark gray).

To test the hypothesis that the PQC capacity decreases in AD, we performed a meta-analysis of the proteomics data provided by the Unwin laboratory, which is freely available at http://www.dementia-proteomes-project.manchester.ac.uk/Proteome/Search (Xu et al., 2019; Figure 1A). This study reveals a quantitative overview on protein levels of nine AD brains and nine healthy age-matched control brains, separated per brain region. The six distinct brain regions studied range from mostly unaffected in AD (cerebellum) to mildly affected (motor cortex and sensory cortex) and strongly affected (hippocampus, entorhinal cortex and cingulate gyrus) (Smith, 2002; Xu et al., 2019; Figure 1B). We analyzed 106 distinct proteins involved in different pathways of the PQC to obtain a comprehensive overview of protein levels of components of the PQC in AD (Figure 1A). AD levels are represented as fold-change compared to control brain.

We assessed the significance of increase or decrease in protein levels for each PQC component considering the following: (i) a change relevant to the disease is likely to show a pattern reflecting the extent of how severely brain regions are affected by AD. (ii) The extent of the alteration in protein levels, as chaperones act stoichiometrically. (iii) The false-discovery rate (FDR), indicating an estimate on variability of the data. An FDR of e.g., 1% indicates the likelihood that in 99 out of 100 cases there would be a true positive difference, for an FDR of 10% this would drop to 90 out of 100 cases. As we aim to evaluate alterations for individual components of the PQC we aim to provide a balanced evaluation for each analyzed protein taking these criteria into account.

### Neuronal and Glial Cells

**FIGURE 2:**
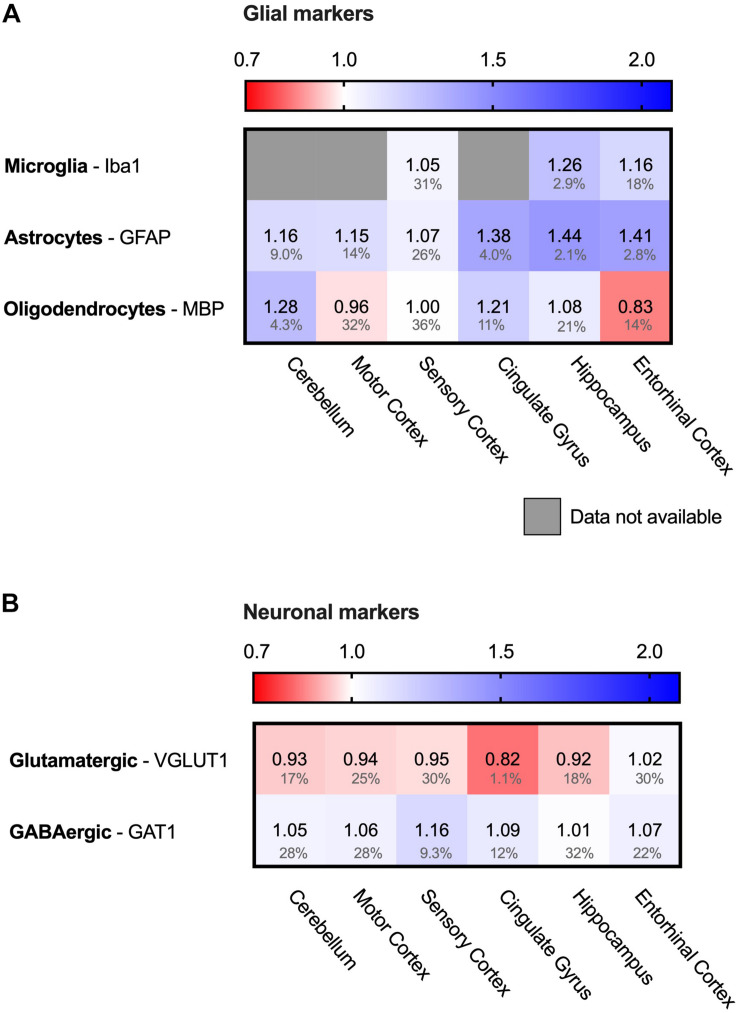
Both neuronal and glial cells present in analyzed sample. Heat map of neuronal and glial protein markers in different brain regions, differently affected in Alzheimer’s Disease. Numbers represent the fold change in AD brains versus controls, indicated per brain region (blue gradient; white, no change; decrease, red gradient). Gray boxes represent unavailable data. FDR values are indicated as percentage. **(A)** Protein markers for all glial cells are present in all six brain regions, indicating presence of these types of cells in the analyzed sample. **(B)** Protein markers for glutamatergic (excitatory) neurons and GABAergic (inhibitory) neurons, indicating presence of both type of neurons in analyzed brain tissue.

Brain tissue consists of multiple cell types, which either are neuronal cells or glial cells. While neuronal degradation is at the heart of the disease, also glial cells are implied to have a role in AD (Dzamba et al., 2016). To assess whether the database allows conclusions on the PQC system in all different cell types, we analyzed for the presence of cell-type specific protein markers (Figure 2). For glial cells at least one specific protein markers is present for each of the three types of glial cells; IBA1 (AIF1) for microglia, GFAP for astrocytes and MBP for oligodendrocytes (Figure 2A), indicating that all of them are still present in AD brain (Imai et al., 1996; Pekny and Pekna, 2004; Nawaz et al., 2013). For microglia and astrocytes we do observe a pattern related to disease progression with relatively small FDR values. Microglia marker levels are increased with 16–26% in hippocampus and entorhinal cortex, and astrocytes increase with 38–44% in all three affected brain regions, possibly indicative of an overrepresentation of these type of cells in affected brain regions. For neurons, we also assessed the presence of markers for the brain’s most prominent excitatory and inhibitory neurons; VGLUT for glutamatergic and GAT1 (SLC6A1) for GABAergic neurons (Borden, 1996; Fremeau et al., 2001). Both proteins were present in all six brain regions (Figure 2B), indicating that these neurons are also represented in the database.

### Levels of Tau Are Not Affected in AD

**FIGURE 3:**
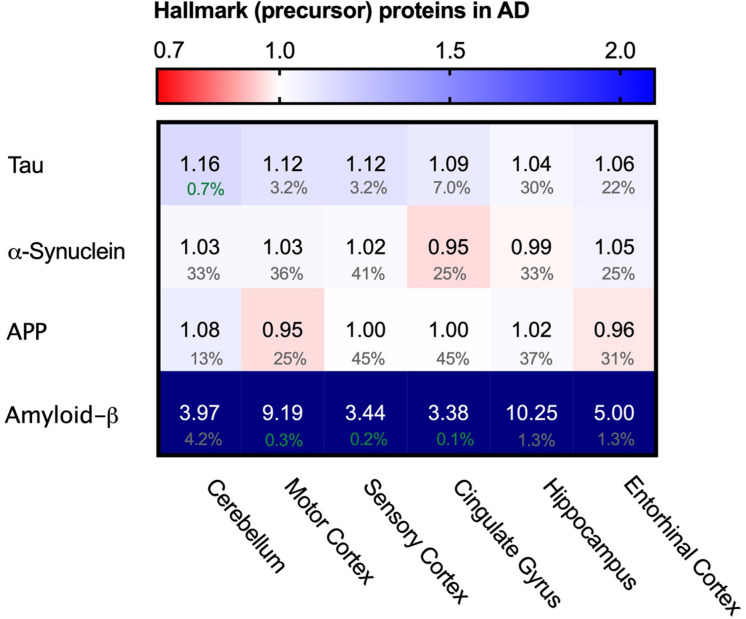
Levels of Tau and α-synuclein not affected in Alzheimer’s brain. Heat map of protein levels in different brain regions, differently affected in Alzheimer’s Disease. Numbers represent the fold change in AD brains versus controls, indicated per brain region (blue gradient; white, no change; decrease, red gradient). Gray boxes represent unavailable data. FDR values are indicated as percentage. Tau levels remain largely unaffected with only minor increase in some brain regions, whereas α-synuclein remains unaffected in all brain regions. Amyloid-ß is strongly increased in all affected brain regions. Increase however does not correlate with affectedness of brain regions.

As Tau fibrils are a major hallmark of Alzheimer’s Disease, we wondered whether the appearance of tangles may be reflected in potentially higher levels of Tau (MBPT) in AD brains. Analysis of Tau protein levels between AD brains and control, however, showed no major differences in protein levels in any of the brain regions (Figure 3). This indicates that Tau fibril formation is not driven by an increase total Tau levels.

Besides intracellular accumulation of Tau protein, extracellular accumulation of amyloid-β is another hallmark of AD (Serrano-Pozo et al., 2011). As amyloid-β is a proteolytic product of amyloid precursor protein (APP) we analyzed levels of both APP and amyloid-ß peptide. Levels of APP do not change, whereas levels of amyloid-ß are increased in all brain regions and even show an astonishing 10-fold increase in the hippocampus (Figure 3), which is in line with previous reports (Seyfried et al., 2017). The methodology does not allow, however, to distinguish between Aβ1-40 and Aβ1-42 – of which the latter is thought to be the aggregation-prone peptide. The increases of amyloid-β peptide do not correlate with affectedness of the brain regions, nor is it restricted to AD brains alone as control brains also show great inter-patient variability of amyloid-β peptide levels (Supplementary Figure S1), which in line with the observation how individuals can remain cognitively normal despite presence of Aβ plaques or neurofibrillary tangles (Sperling et al., 2011).

We also looked into the levels of α-synuclein (SNCA). α-synuclein is an aggregating protein in Parkinson’s Disease and Fronto-Temporal Dementia but is also involved in AD via crosstalk with Tau in promoting each other’s aggregation (Attems and Walker, 2017). When analyzing protein levels of α-synuclein we could not identify notable differences in the distinct brain regions (Figure 3). Together, there are no noteworthy differences in the levels of two major proteins that aggregate in disease. This suggests that the key difference in the AD brain is not related to changes in the levels of the aggregating proteins themselves. Therefore, we set out to investigate whether disturbance of the PQC network, which controls and prevent protein aggregation in healthy neurons, may be a hallmark of neurons in AD.

### Chaperonins Do Not Alter in AD

**FIGURE 4:**
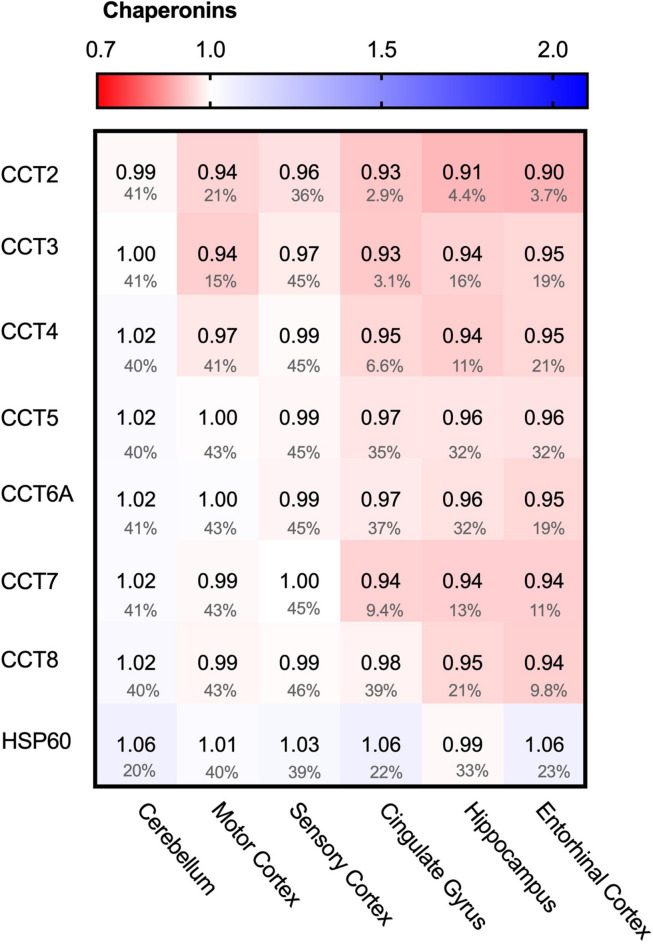
Chaperonin levels do not alter in AD. Heat map of chaperonins in different brain regions. Numbers represent the fold change in AD brains versus controls, indicated per brain region (blue gradient; white, no change; decrease, red gradient). Gray boxes represent unavailable data. FDR values are indicated as percentage. None of the TriC/CCT subunits show notable differences in protein levels. Mitochondrial HSP60 levels also remain fairly constant in all brain regions in AD.

We started the analysis of the PQC system with the largest folding machine in the metazoan cytosol, the ATP-dependent HSP60 chaperone family, also known as chaperonins (Ansari and Mande, 2018). The chaperonin TRiC/CCT is associated with protein aggregation in disease, in particular in Huntington’s Disease, where it can bind to specific subunits of the huntingtin protein and modulate its aggregation (Spiess et al., 2006; Tam et al., 2009). We wondered whether chaperonin levels are affected in AD and analyzed the levels of the seven TRiC/CCT subunits in the cytoplasm and HSP60 in mitochondria. None of the TRiC/CCT subunits showed notably altered levels in the Alzheimer brain, ranging from only 10% for CCT2 in the entorhinal cortex to no change at all in the motor cortex for CCT7 (Figure 4). Similarly, HSP60 levels did not differ between Alzheimer and control brains, as values change from a minor decrease of 1% in the hippocampus to a slight 6% decrease in the cerebellum (Figure 4). Alterations in chaperonin levels thus do not to play an important role in AD.

### Decrease of All Hsp90 Paralogs in AD

**FIGURE 5:**
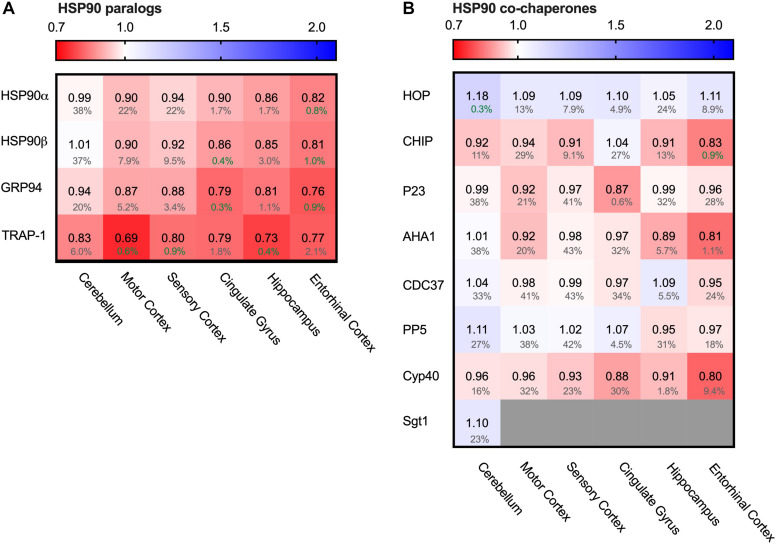
All HSP90 paralogs decreased in AD. Heat map of HSP90 paralogs and it’s co-chaperones in different brain regions. Numbers represent the fold change in AD brains versus controls, indicated per brain region (blue gradient; white, no change; decrease, red gradient). Gray boxes represent unavailable data. FDR values are indicated as percentage. **(A)** All HSP90 paralogs show a decrease in protein levels, with the strongest decrease in mitochondrial HSP90, TRAP1. The cerebellum, which is the most unaffected brain region in AD, appears to be mostly unchanged, except for TRAP1 levels. **(B)** Co-chaperones of HSP90 show no strong differences between control and AD brain.

One of the major chaperone systems controlling Tau is the evolutionary conserved Hsp90 machinery (Karagöz et al., 2014). To see if Hsp90 levels are affected in AD brain, we compared in the database the levels of all four Hsp90 paralogs. Strikingly, all Hsp90 paralogs were decreased in all brain regions affected in AD (Figure 5A).

Of all Hsp90s, the mitochondrial TRAP1 showed the most pronounced decrease in Alzheimer brains, with a drastic decrease of 31% in the motor cortex. The other AD affected brain regions also showed severe reduction in TRAP1 levels, such as a decrease of 27% in the hippocampus and 21% in cingulate gyrus. For the cytosolic house-keeping paralog HSP90β, levels decreased with 18% in the entorhinal cortex in AD compared to control, and heat-shock inducible HSP90α showed a similar reduction in the same brain region (19%). Interestingly, for all paralogs the levels in the cerebellum do not show notable changes in AD, which is the brain region least affected in AD. The strength with which HSP90 levels decrease in other regions appear to correlate with level of affectedness, which makes it likely that the decrease of Hsp90 levels in the more disease-affected brain regions is Alzheimer-related.

To place the relative reduction levels into context, it is important to realize that the changes documented in the database represent the averaged protein levels in the entire tissue. Some cells may be severely depleted while others remain fairly unaffected. This makes it likely that actual changes in Hsp90 levels in AD-affected cells are more drastic than reflected in the percentage values in the database.

The function of cytoplasmatic Hsp90 is specified by a plethora of co-chaperones. We looked, therefore, also into protein levels of several co-chaperones. We started from a canonical set of Hsp90 co-chaperones functionally compared in a comprehensive study (Sahasrabudhe et al., 2017; Figure 5B). In contrast to the Hsp90s, alterations in co-chaperone levels were only minor (4% decrease for p23 (PTGES3) in entorhinal cortex and 8% decrease for Aha1 ((AHSA1) in motor cortex) and were also accompanied by high FDR rates, which render them insignificant. TPR co-chaperones like HOP and CHIP remain relatively unaffected and show no correlation with disease progression. Thus, although all Hsp90s itself are depleted in AD, its regulatory network is not derailed.

### HSP70 but Not HSC70 Enhanced in AD

**FIGURE 6:**
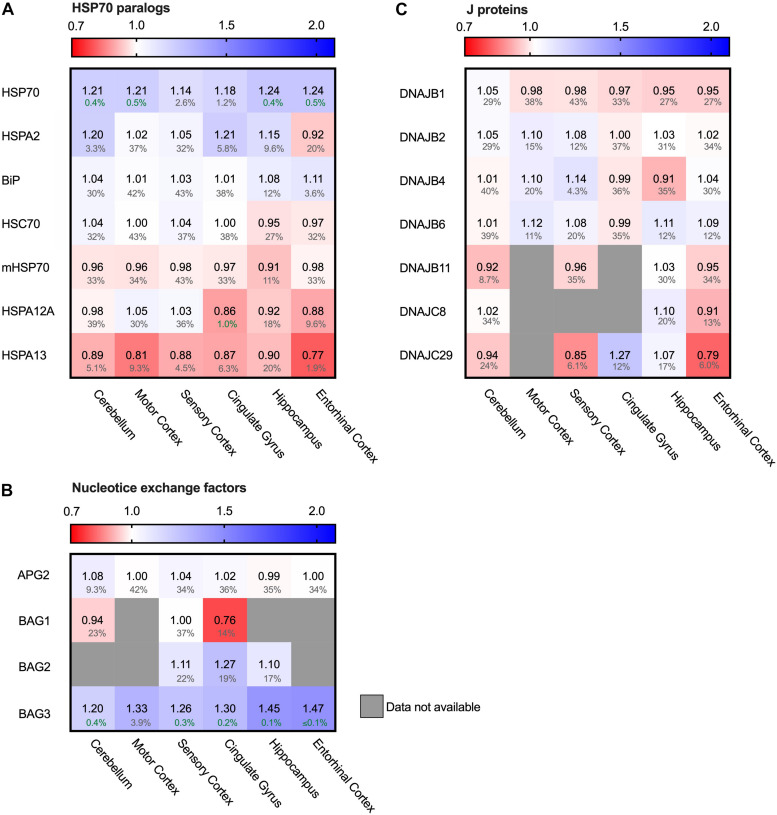
Only heat-shock inducible HSP70 strongly increased in AD. Heat map of paralogs of HSP70 and its co-chaperones in different brain regions. Numbers represent the fold change in AD brains versus controls, indicated per brain region (blue gradient; white, no change; decrease, red gradient). Gray boxes represent unavailable data. FDR values are indicated as percentage. **(A)** Heat shock inducible HSP70 is strongly upregulated in all brain regions, whereas all other paralogs of HSP70 remain constant. **(B,C)** All co-chaperones of HSP70 are unaffected, except for BAG3. BAG3 is strongly increased in all brain regions. J-proteins do not show a general tendency in increase or decrease. DNAJC29 is decreased in sensory cortex and entorhinal cortex, but a high false discovery rate renders these changes not significant.

Hsp90 depletion is known to upregulate HSF1 (Zou et al., 1998) which is considered to be the master regulator of the heat shock response and is involved in the regulation of Hsp70 expression. Next to Hsp90, Hsp70 is the other conserved ATP-dependent chaperone family that is present in all folding compartments. Hsp70 acts upstream of Hsp90 in the early stage of the folding cascade and is known to interact with Tau (Dickey et al., 2007). We evaluated the levels of seven out of twelve Hsp70 paralogs (Radons, 2016; Serlidaki et al., 2020). The five paralogs not present in the analysis did not appear in the database. All these paralogs are constitutively expressed, except for the cytosolic heat-shock inducible HSP70 (HSPA1), which is under the regulation of HSF1. Remarkably, heat-shock inducible HSP70 was strongly increased in AD brain of all brain regions affected in disease (e.g., 24% in the hippocampus and entorhinal cortex and 18% in cingulate gyrus) - including the relatively unaffected cerebellum - whereas the constitutively expressed paralogs remained constant (Figure 6A). Of those, only HSPA13 does show slightly decreased levels in all brain regions (at high FDR values), with a more pronounced decrease in the entorhinal cortex (reduction by 23% in AD at an FDR value of 1.9%). HSPA12A shows limited decrease in the cingulate gyrus. In contrast to the HPS90 paralogs, the observed changes for HSP70 paralogs do not show a correlation with level of affectedness of the brain regions. In conclusion, the major change for the Hsp70s is reserved to the stress-inducible HSP70. As HSP70 is under regulation of HSF1, it is involved in the stress-response of the cell and its upregulation may indicate derailment of the cellular stress response.

### HSP70 Efflux Derails Toward Autophagy

Hsp70s are functionally dependent on co-chaperones. We looked into a subset representing the most prominent J-proteins and Nucleotide Exchange Factors. J-proteins stimulate the Hsp70 ATPase and act as substrate targeting factors for Hsp70s, thereby controlling the influx into this chaperone system. We evaluated the protein levels of J-proteins associated with neurodegenerative diseases (Kampinga and Craig, 2010). Notably, we did not observe major differences for any of the J-proteins (Figure 6C). The most notable change was an increase of 14% for DNAJB4 in the sensory cortex, but this co-chaperone did not show a general pattern in relation with AD affected brain regions. Overall, the levels of J-proteins do not show prominent differences between Alzheimer and control brains, which makes it unlikely that control of substrate influx into the Hsp70 system is disturbed in AD.

After ATP hydrolysis of Hsp70, the subsequent replacement of ADP by ATP releases the substrate protein. Nucleotide Exchange Factors (NEFs) trigger this exchange, thereby controlling environment and conditions of release and resetting the Hsp70 system (Mayer and Gierasch, 2019). Thus, NEFs regulate substrate efflux, triaging the fate of the substrate after its release from Hsp70, including refolding, disaggregation and degradation either by the proteasome or by autophagy. A key NEF for folding and disaggregation is APG2 (HSPA4) (Bracher and Verghese, 2015). It contributes to the disaggregation capacity of Hsp70 for aggregates of Tau, α-synuclein and huntingtin (Gao et al., 2015; Ferrari et al., 2018; Scior et al., 2018; Nachman et al., 2020). In AD brains however, its levels remained unaffected (Figure 6B). Strikingly, levels of another NEF, BAG3, are strongly elevated in AD brain (Figure 6B). Interestingly, BAG3 has a specific role in chaperone assisted selective autophagy by forming a multi-chaperone complex for ubiquitylation and sequestration of its client protein and facilitates in the substrate engulfment by the autophagosome (Klimek et al., 2017). Its expression is part of the cellular stress response and controlled by HSF1 (Franceschelli et al., 2008). Levels of its family members BAG1 and BAG2 were not decisively altered (Figure 6B). The strong upregulation of BAG3 in AD brains, in striking contrast to its family members, implies a change in the efflux control of the Hsp70 machinery towards activation of the autophagy pathway.

### Upregulation of Several sHSPs

**FIGURE 7:**
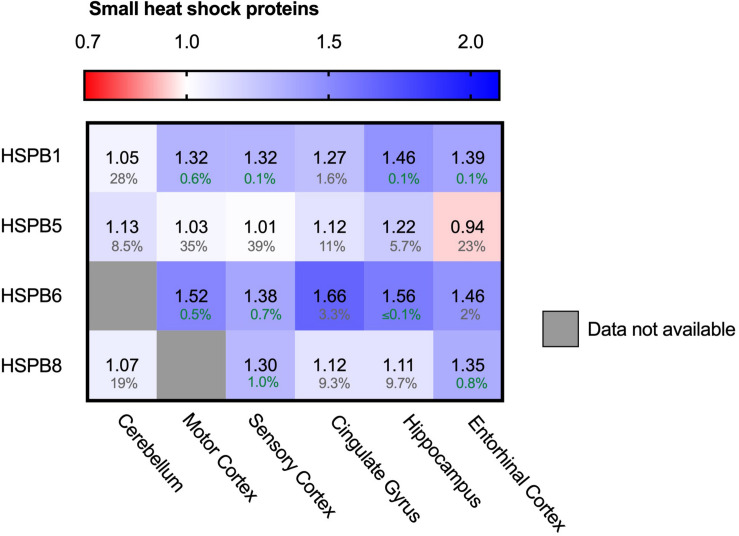
Neuronally expressed sHSPs upregulated in AD brain. Heat map of sHSPs in different brain regions. Numbers represent the fold change in AD brains versus controls, indicated per brain region (blue gradient; white, no change; decrease, red gradient). Gray boxes represent unavailable data. FDR values are indicated as percentage. HSPB1 and HSPB6 are strongly increased in all brain regions, HSPB5 and HSPB8 show only minor differences accompanied by high FDRs.

In contrast to the ATP-driven Hsp70 and Hsp90 systems, small heat shock proteins (sHSPs) are the largest ATP-independent class of chaperones. They act early-stage on hydrophobic stretches of their substrates, possibly upstream of the ATP-dependent Hsp70-Hsp90 chaperone cascade (McHaourab et al., 2009; Bakthisaran et al., 2015; Haslbeck and Vierling, 2015; Zwirowski et al., 2017; Morán Luengo et al., 2019). Out of 10 mammalian sHSPs, four family members are expressed in neurons and play a role in neurodegenerative diseases: HSPB1, HSPB5, HSPB8 and to a lower extend, HSPB6 (Quraishe et al., 2008; Webster et al., 2019). HSPB1 and HSPB6 both show a strong increase in all brain regions (Figure 7). Similar trends can be observed for HSPB5 and HSPB8, although they are accompanied by slightly higher FDR values and may thus not be fully representative. HSPB1 levels increased from only 5% in the unaffected cerebellum up to 46% in the hippocampus. For HSPB6 levels increased even further up to 66% in the cingulate gyrus. sHSPs co-operate with HSP70 and BAG3, the two other chaperone components also strongly increased in AD brain (Figure 5; Rauch et al., 2017). The sHSP-HSP70-BAG3 system channels its substrate towards chaperone assisted selective autophagy (Stürner and Behl, 2017). Thus, within the entire cellular chaperone network only the pathway leading towards autophagy is upregulated in AD.

### Alterations in Autophagy Markers in Alzheimer Brains

**FIGURE 8:**
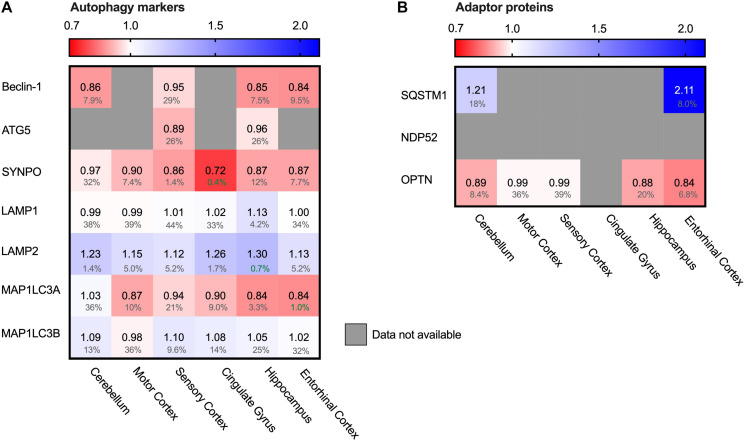
Chaperone-mediated autophagy adaptor SQSTM1 increased in AD Heat map of autophagy markers and their adaptor proteins in different brain regions. Numbers represent the fold change in AD brains versus controls, indicated per brain region (blue gradient; white, no change; decrease, red gradient). Gray boxes represent unavailable data. FDR values are indicated as percentage. **(A)** General autophagy-markers do not show any significant difference, except for MAP1LC3A in the strongest affected brain regions (Hippocampus, Entorhinal Cortex and Cingulate Gyrus). Observed differences in other brain regions are non-representative due to high false discovery rate. **(B)** for the autophagy adaptor proteins, the protein SQSTM1 is increased in both brain regions for which data are available.

Autophagy is the degradation pathway dedicated to protein aggregates and dysfunctional cellular compartments (Finley, 2009). In particular, selective macro-autophagy targets such aberrant components and engulfs them in double membrane compartments termed autophagosomes. Subsequent fusion with lysosomes allows enzymatic degradation (Stolz et al., 2014). Protein aggregates can be cleared by selective autophagy - a process referred to as aggrephagy – and could possibly be of relevance in AD. LC3 proteins are early-stage markers for autophagy and are important for the autophagosome formation. Map1LC3A levels were decreased in AD brains (Figure 8A, 16% in entorhinal cortex), indicating impairment in this process. Several adaptor proteins are known for autophagy, with different adaptor proteins specific for individual autophagy pathways. Adaptor protein SQSTM-1 is *key* marker for aggrephagy (Zaffagnini et al., 2018) and showed an extreme increase in protein levels in the brain region most affected in AD; the entorhinal cortex (111%, Figure 8B). Increased levels of SQSTM-1 are indicative of impaired autophagy (Bjorkoy et al., 2005) implying that this process is impaired in AD. More specifically, increase of SQSTM-1 is likely indicative of recruitment of SQSTM-1 mediated aggrephagy in AD, as other autophagy adaptors, such as OPTN, are not meaningfully altered in AD.

### Proteasomal Degradation System Remains Largely Unaffected

**FIGURE 9:**
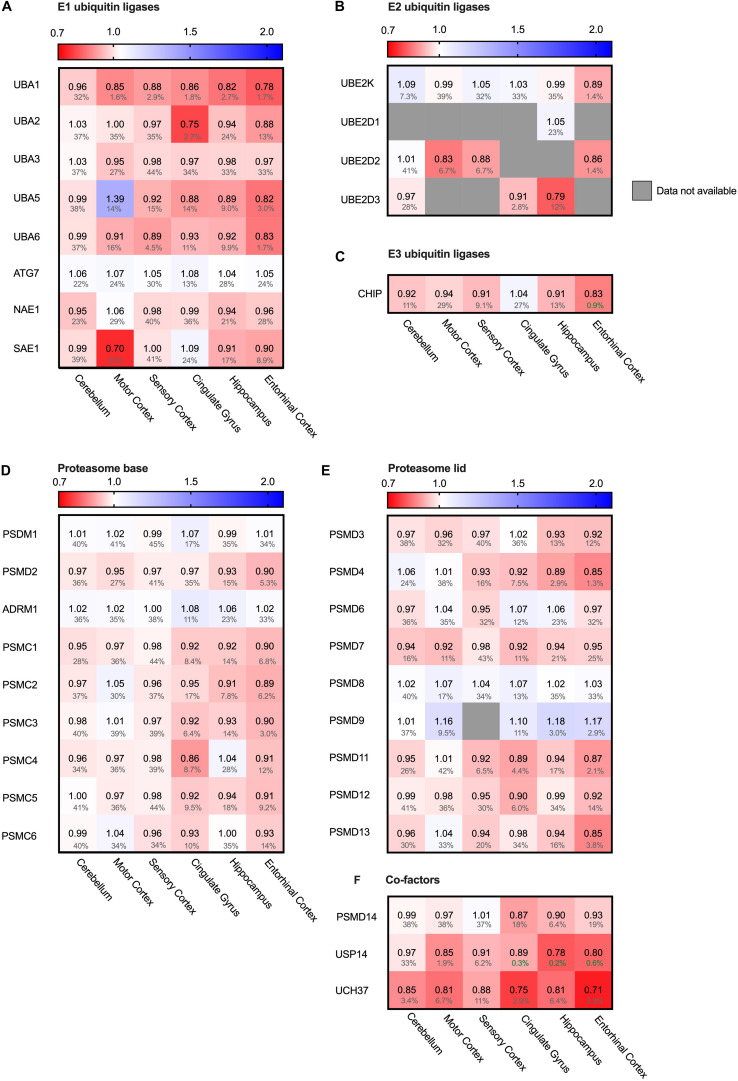
Ubiquitin ligases largely unaffected in AD. Heat map of components of the ubiquitin ligases in different brain regions. Numbers represent the fold change in AD brains versus controls, indicated per brain region (blue gradient; white, no change; decrease, red gradient). Gray boxes represent unavailable data. FDR values are indicated as percentage. **(A–C)** E1, E2 and E3 ubiquitin ligases do not show any notable difference in protein levels in AD affected brain regions. **(D–F)** Components of the base and lid of the proteasome do not show any notable difference in protein levels in any of the brain regions. Some co-factors of the proteasome, such as Usp14 and Uch37, are slightly decreased in AD brain.

The main cytosolic degradation pathway for many processes is the ubiquitin-proteasomal machinery, including for the in Alzheimer accumulating protein Tau. A cascade of E1, E2 and E3 ligases targets proteins for degradation by flagging them with a poly-ubiquitin chain (Zheng and Shabek, 2017). When analyzing levels of several different E1, E2 and E3 ubiquitin ligases in the Alzheimer brain, we did not note significant differences. For small subset of ubiquitin ligases, we did observe minor decreases in the fold change accompanied by high FDR values, rendering them insignificant (Figures 9A–C). E1 ubiquitin ligase UBA1 for instance was decreased by 22% in the entorhinal cortex and SAE even by 30% in the motor cortex (Figure 9A). However, high FDR values mark them as likely outliers.

Next, we turned from the targeting cascade to the degradation machinery itself and analyzed the different subunits of the proteasome. The proteasome is build-up of two complexes: a four-ring core component and one or two regulatory lid structures (Coux et al., 1996). Neither for the proteins constituting the lid or base of the proteasome we noted differences in protein levels (Figures 9D,E). Interestingly, however, two of the three metazoan de-ubiquitinating enzymes associated with the proteasome (de Poot et al., 2017), USP14 and UCH37 (UCHL5), showed decreased levels throughout all brain regions, ranging from 13 to 29% (Figure 9F). These factors trim the ubiquitinated substrate on the proteasome, slowing down proteasomal degradation (Lam et al., 1997). Inhibition of these factors upregulates proteasomal activity (Lee et al., 2011). Decreased levels of these co-factors in AD brains could thus indicate an increase of proteasomal flux.

### Hsp90 Only Stress System Affected in Mitochondria

**FIGURE 10:**
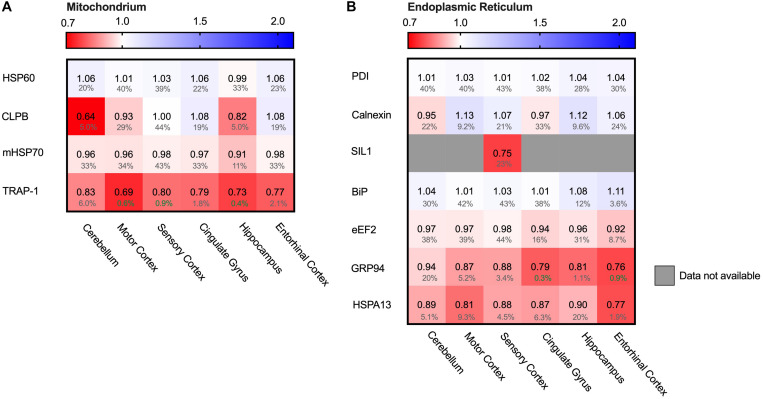
HSP90 paralog TRAP1 strongly decreased in AD. Heat map of mitochondrial and ER chaperones in different brain regions. Numbers represent the fold change in AD brains versus controls, indicated per brain region (blue gradient; white, no change; decrease, red gradient). Gray boxes represent unavailable data. FDR values are indicated as percentage. **(A)** The HSP90 paralog, TRAP1, shows a strong decrease in protein levels in AD. Other mitochondrial chaperones, including mitochondrial HSP70, HSP60 or CLPB, do not show any notable changes in protein levels in AD. **(B)** Strong decrease in protein levels of GRP94, the HSP90 ER paralog. Other components remain fairly unaffected. The decrease in SIL1 protein level most likely is not representative because of high false discovery date.

Protein folding is a process not restricted to the cytosol, but also occurs in other cellular compartments such as the mitochondria. Mitochondria are essential for energy supply, but also play a key role in activation of cellular apoptosis and thus cellular degeneration. We therefore analyzed four mitochondrial chaperones (Figure 10A). HSP60 (HSPD1), CLPB and mt-HSP70 (HSPA9) did not show major differences in protein levels. The Hsp90 paralog TRAP1 (HSP90B2P) is thus the only mitochondrial chaperone strongly decreased in all brain regions. This downregulation of TRAP1 reflects a specific pathway, as a general stress response would most likely affect levels of other chaperones in mitochondria as well.

### Endoplasmic Reticulum Chaperones Do Not Shows Remarkable Alterations

The endoplasmic reticulum (ER) is a cellular compartment important in protein production and folding. ER stress leads to the activation of the Unfolded Protein Response (UPR), implied to be upregulated in AD in the hippocampus and entorhinal cortex (Hoozemans et al., 2005; Cornejo and Hetz, 2013). The ER paralog of Hsp90 - Grp94 (HSP90B1) - does show a strong decrease, ranging from a minor 6% in the mostly spared cerebellum, up to an astonishing 23% in the highly affected entorhinal cortex (Figure 10B). Individual levels of the main luminal Hsp70, BiP (HSPA5), shows no major changes. Data on levels of the Hsp110 SIL1, NEF for BiP, however, are mostly unavailable, with the exception of the sensory cortex where SIL1 shows a decrease of 25%. However, this is not representative, as this finding is restricted to only one brain region and accompanied by a high FDR value of 23%. Other UPR markers, such as PDI (P4HD) and Calnexin (CANX), show no major differences in any of the brain regions in AD. As there is no general increase of UPR specific markers we conclude that the Alzheimer brain is not characterized by a fully activated UPR.

## Discussion

**FIGURE 11:**
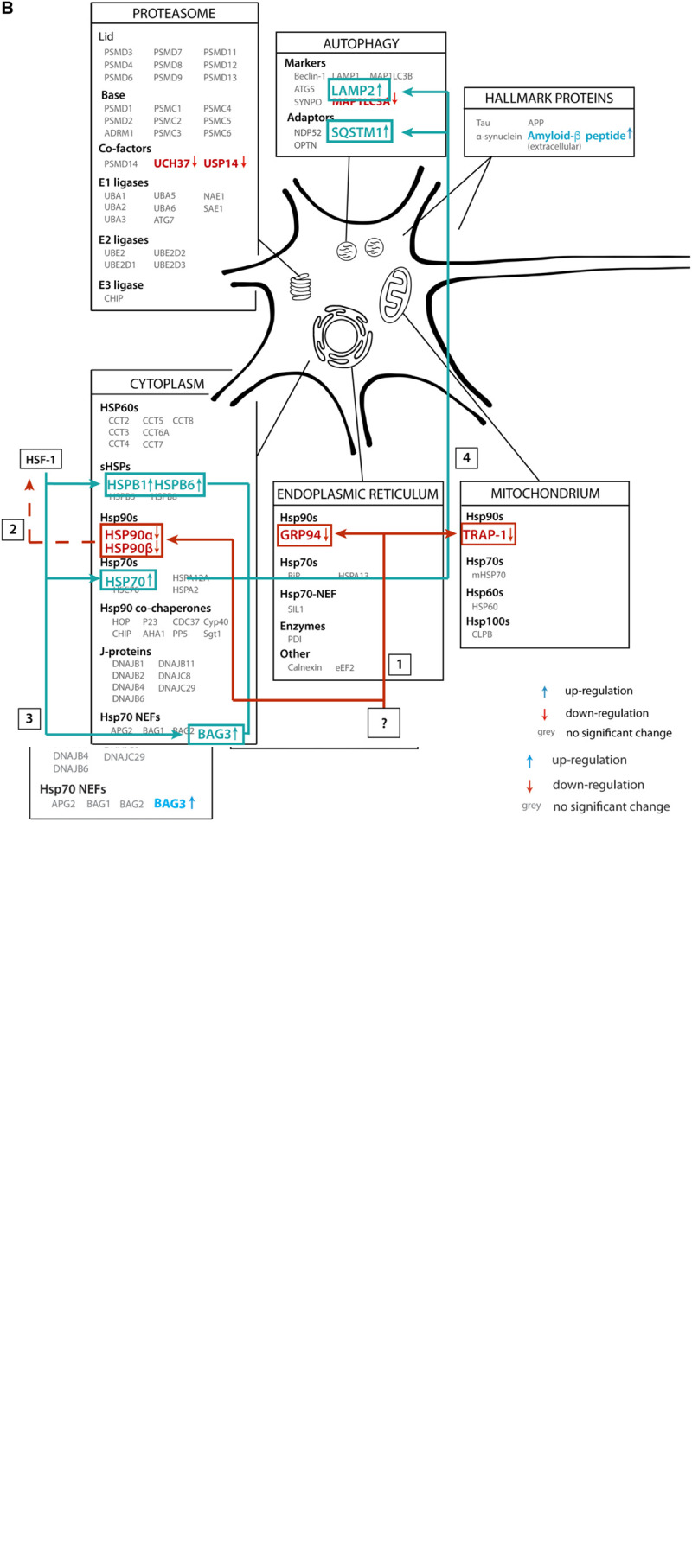
Illustrative overview of main findings. **(A)** Schematic representation of the alteration of protein levels in brain tissue in our analysis. Proteins are sorted per compartment and classification. Proteins represented in gray are not significantly altered in AD brains, proteins in blue are upregulated and proteins in red are downregulated. **(B)** Functional connection of PQC factors affected in AD. An unknown event ‘?’ causes downregulation of all HSP90 paralogs (1), which may lead to activation of transcription of heats shock genes, possibly via HSF1 (2), leading to subsequent upregulation of HSP70, BAG3 and sHSPs (3). These factors then trigger activation of chaperone assisted selective autophagy (4).

In this paper we aimed to reveal systematic changes in the PQC network in Alzheimer brains (Figure 11A). We analyzed data from a proteomics database obtained from 18 individuals, of which nine AD cases and nine control. Interestingly, the majority of players in the field remain unaffected, but a subset of the PQC components is either up- or downregulated in AD. Levels of all Hsp90 paralogs in cytoplasm, mitochondria and ER are strongly decreased. In contrast, a chaperone system consisting of heat shock inducible HSP70, its NEF BAG3 and several sHSPs show remarkable increases in protein levels (Figure 11A).

Our PQC analysis at the protein level revealed interesting differences compared to transcriptome studies of AD brains (Brehme et al., 2014). At the RNA level, 135 PQC genes are significantly altered, up to threefold. Interestingly, at the protein level the changes are less extreme and much more targeted to specific processes. While at RNA level, e.g., many Hsp70 (co-) chaperones such as a number of J-domain proteins are repressed, they are largely unaffected at the protein level. The small number of specific (co)chaperones upregulated at the protein level are also overexpressed in AD, including HSP70, BAG3 and HSPB8 (Figures 6, 7; Brehme et al., 2014; Fu et al., 2019). Consistently, Hsp90 genes are repressed and exhibit reduced protein levels (Figure 5; Brehme et al., 2014). These proteome changes are stronger in tissues more severely affected in AD. It would be intriguing to compare this to tissue-specific data at the transcriptome level should such data become available. Overall, compared to adaptations of the transcriptome, changes at the protein level are more targeted to a limited number of key factors, which may be related to translational control of PQC factors (Harding et al., 2000).

### Neurons Versus Glial Cells

Our findings are based on analyzing the Alzheimer Disease Proteome database from the point of view of the PQC system (Xu et al., 2019). Thus, the accuracy of our conclusions depends on the quality of the data provided. While the data specify protein levels per brain region, they do not resolve different cell types with these regions. The brain material from which the samples are taken consists of both glial and neuronal cells, and protein markers of both types of cells were present in the analyzed tissue. Therefore, both neurons and glial cells may contribute to the measured protein levels (Figure 2). In an extreme although unlikely case opposing changes in protein levels in both cell types may even cancel each other out. It is evident from our analysis, however, that the deviations in the Alzheimer tissue reflect a specific adaptation of a particular cellular stress pathway, raising the question which cell type may be affected most.

There are three options: (i) The stress response is exclusive for neurons; (ii) it is exclusive for glial cells; and (iii) the response takes place in both neuronal and glial cells. There is conflicting evidence whether specific sHsps are expressed more in neuronal or glial cells (Wilhelmus et al., 2006; Björkdahl et al., 2008; Schwarz et al., 2010). HSPB1 and HSPB5 are upregulated in neurodegenerative diseases, especially in reactive glial cells and HSPB8, together with BAG3, is upregulated in astrocytes (Seidel et al., 2012). Both microglia and astrocytes are activated in the neuroinflammatory response and implied to contribute to AD pathology (Stürner and Behl, 2017; Kaur et al., 2019). However, as the neurons are the cells that are suffering from intracellular protein aggregation, it seems likely that the observed changes in protein quality control represent a response inside neurons as well. Given that in the cortex, which is most affected in Alzheimer, glial cells outnumber neurons by 4:1, the alterations in PQC level would underestimate the reaction at neuronal level. In contrast, the second alternative would imply that the neurons would be unable to respond to the stress caused by intracellular aggregation of Tau and instead the glial cells have elevated levels of stress proteins. In contrast, activation of the innate immune response, as noted by the Unwin group, may reflect activation of microglia and subsequent neuroinflammatory response (Xu et al., 2019). Experiments with cell-type specific read out will ultimately be needed to decide between the three possibilities.

### Stress Response

HSP70, BAG3 and the sHSPs have in common that they are all under HSF1 regulation (Lindquist, 1986; Mathew and Morimoto, 1998; Bjork and Sistonen, 2010). HSF1 levels escaped detection in the proteomics screen, both in AD and control tissue. Regulatory proteins such as HSF1 are present in low levels, making detection more challenging. However, it is possible to monitor levels of heat shock proteins such as HSP70, BAG3 and sHSPs to draw conclusions on activation of the stress response. These three components can form a chaperone-complex in the autophagy-pathway, targeting their substrates toward lysosomes for degradation (Stürner and Behl, 2017). A decrease in HSP90 can contribute to the upregulation of HSF1, as inhibition of HSP90 triggers the heat shock response (Zou et al., 1998). Upon upregulation of HSF1, there is an elongated occupation of HSF1 on the HSP70 gene resulting in increased levels of HSP70 (Do et al., 2015; Shapiro et al., 2015). Downregulation of HSP90 and upregulation of sHSP-HSP70-BAG3 may thus be functionally linked in Alzheimer neurons.

### Degradation

Besides activation of HSF1, a decrease in HSP90 may also have a direct effect on Tau turnover. Inhibition of HSP90 promotes proteasomal degradation of monomeric phosphorylated Tau (Dickey et al., 2007). If a decrease in monomeric Tau degradation is the result of decreased levels of HSP90, this may lead to an accumulation of phosphorylated monomeric Tau, which can give rise to fibril formation. Proteasomal degradation of oligomeric or fibrillar Tau however may not occur, though, as the narrow pore of the proteasome restricts the maximum size of the substrate to be degraded, precluding degradation of protein oligomers or aggregates (Williams et al., 2006).

The other important degradation pathway in AD is the autophagy-lysosome pathway (Wong and Cuervo, 2010). One of the autophagy pathways - macro-autophagy – is upregulated under normal aging conditions (Gamerdinger et al., 2009). The BAG3/BAG1 ratio increases during aging, which is indicative of sequestering toward the autophagy versus proteasomal degradation. BAG1 and BAG3 are both NEFs for HSP70; the HSP70:BAG1 complex targets substrates for proteasomal degradation whereas the HSP70:BAG3 complex in cooperation with sHSPs can sequester aggregates for autophagic degradation (Carra et al., 2008). A further increase of the BAG3:BAG1 ratio in AD brain may reflect an even stronger enhanced autophagic activity.

Interestingly, a strong increase of aggrephagy marker SQSTM-1 as observed in AD (Figure 8B) is indicative of impaired autophagy. Under normal conditions, SQSTM-1 is, together with cargo, cleared by the lysosomes (Bjorkoy et al., 2009). Possibly these observations in AD brain relate to protein aggregates triggering co-operation of sHSPs, HSP70 and BAG3, thereby enhancing the macro-autophagy pathway. However, an immense influx of protein aggregates may overload the autophagic pathway, which in turn may enhance SQSTM-1 levels to prevent clogging of the system.

### Hen and Egg Question

One of the most stunning questions is why all paralogs of Hsp90 are decreased in AD brains and thus what is upstream of the Hsp90s that may trigger this. In other words, is the decrease in Hsp90 levels cause or consequence? The answer to this question may bring us one step closer to understand to which extent alterations in the PQC network represent a cellular reaction to events causing AD or whether PQC adaptions are at the fundaments of the pathology themselves.

Analysis into the cause of the disease is further complicated by the fact that post-mortem data intrinsically constitute a one-point measurement at a moment the brain ceased to function. Protein levels of the neurons who suffered most from AD cannot be measured, as these neurons already degenerated. The protein levels we study here represent the average in brain regions that contain neurons or glial cells possibly already on their way to death and thus have not reached their end-stage yet. Such cells may represent the *status quo* on the road to derailment. Another point to take into consideration is that the observed alterations may represent adaptive responses from cells that survived neighboring cells dying from neurodegeneration. Thus, alterations in post-mortem material are likely to underestimate the derailment of the PQC in cells on the brink of cell death.

Alterations in the PQC may prepare cells to stand damage caused by protein aggregation. Transcellular signaling may play a role preparing neighboring and still unaffected tissue for upcoming damage (Prahlad et al., 2008; van Oosten-Hawle and Morimoto, 2014). This concept has been established In C. elegans, where a stress response in neuronal cells already triggers a response in somatic cells, leading us to wonder to which extend similar events may occur between various brain tissues in AD. For some proteins – such as Hsp70 – we observed a strong increase in protein levels in the cerebellum, which is the most unaffected brain region in AD. Transcellular signaling may provide an explanation of an activated stress response in yet unaffected brain regions.

Altered protein levels in AD brain are a good indication of pathway derailment in AD, but there is not necessarily a stringent relation between levels and functionality of a protein. The activity of ATP-dependent chaperones for instance depends on a network of regulatory co-chaperones. Notably, the levels of the co-chaperone-network is largely untouched in Alzheimer. Co-chaperones such as Aha1 can colocalize with Tau and had been identified as potentially interesting drug targets, but this is not reflected in altered levels in the AD proteome (Figure 5B; Shelton et al., 2017; Singh et al., 2020). However, alterations in levels of major chaperones such as HSP70 and HSP90 may indeed have functional consequences (Figure 11B). We hope that these correlative findings may inspire further experiments to decipher the functional role of these pathways in the origin of AD.

In summary, our meta-analysis provides an extensive overview of players in the PQC network in AD, revealing that the majority of factors does not differ between an AD diseased aged brain and a ‘normal’ control brain. It is the specific downregulation of Hsp90 chaperones and upregulation of a pathway centering on the stress inducible HSP70 in AD brains which may result in insufficient capacity for the affected neurons to deal with protein aggregation. We conclude that the PQC - including pathways such as the stress response and autophagy – plays a key role in derailment in AD, which makes them interesting targets to interfere with in neurodegeneration.

## Materials and Methods

To give a comprehensive overview of protein expression levels in chaperone networks in AD, we performed a meta-analysis of an online database provided by the Unwin group (Xu et al., 2019). The Alzheimer’s Disease Proteome is a freely accessible database reporting proteomics data of more than 5000 distinct proteins, comparing Alzheimer brains versus age-matched control brains. It provides protein levels of nine AD brains and nine healthy control brains. The AD patient group consisted of both male (5) and female (4) patients aged between 60 and 80 years (average age 70 years, median 73 years), classified as Braak stage IV (1), V (3) or VI (5), exhibiting an amyloid load of 2/3 (2) or 3/3 (7). The control group also consistent of both male (5) and female (4) patients, aged between 61 and 78 years (average age 70 years, median 72 years), classified without Braak stage (8) or Braak stage II (1), exhibiting an amyloid load of 0/3 (8) or 2/3 (1) (Xu et al., 2019). The analysis encompassed protein levels from six brain regions, ranging from mostly unaffected to strongly affected in AD. The hippocampus, cingulate gyrus and entorhinal cortex are brain regions strongly affected in AD, motor cortex and sensory cortex are mildly affected, and the cerebellum remains mostly unaffected. For all distinct proteins the data were based on Low-PH LC-mass spectrometry (Xu et al., 2019). We show patient variability for key examples, indicating that differences between individual AD or control samples are typically smaller than between the two groups (Supplementary Figures S2–S10). The analysis does not allow to differentiate between cell types. In cases of PQC factors for which the commonly used names are not identical to the HUGO names, we added the HUGO names at their first appearance in the text.

The database represents protein levels calculated based on peptide presence and analyzed according to a Bayesian protein-level differential quantification. database Protein levels are indicated as logarithmic fold change value of AD brains compared to the age-matched control brain, averaged for each of the populations. The figures in this paper represent numbers indicating non-logarithmic fold changes. A quality indicator for data diversity are False Discovery Rates (FDR). It assesses the percentage of significant false positive hits. An FDR value of 1% indicates a likelihood of a significant false positive finding of 1%, in case of 5% this likelihood is 5%.

We visualized all protein levels in a heat-map format, along a double gradient color-scheme representing the fold change of averaged levels in AD tissue compared to the control population. Baseline for protein levels (fold change value = 1) was set to white, no change of levels in AD compared to control brain. All changes in protein levels were indicated by increasing darkness in a linear color gradient (blue, fold change values > 1; red, fold change values < 1). The double gradient ranged from 0.64 up to 2.1, covering alterations of all analyzed PQC factors. In case some protein level data were unavailable in specific brain regions, boxes are colored gray.

Fold changes of 1.2 or 0.8 reflect changes in protein levels of 20%. Only a small number of proteins show such strong changes, and they were always accompanied by low FDR values. For changes smaller than 20% interpretation requires a closer look at other parameters, such as whether the tissue-specific pattern matches the known AD distribution and the significance of the FDR values.

## Data Availability Statement

Publicly available datasets were analyzed in this study. This data can be found here: http://www.dementia-proteomes-project.manchester.ac.uk/Proteome/Search.

## Author Contributions

MK and SR conceived the study, analyzed the data, and wrote the manuscript. MK gathered data and made figures. All authors contributed to the article and approved the submitted version.

## Conflict of Interest

The authors declare that the research was conducted in the absence of any commercial or financial relationships that could be construed as a potential conflict of interest.
