# Supplementary material for: Alzheimer Cells on Their Way to Derailment Show Selective Changes in Protein Quality Control Network
Source: Front Mol Biosci. 2020 Nov 20;7:214. doi: 10.3389/fmolb.2020.00214 (PMC7715003; doi:10.3389/fmolb.2020.00214)
Supplement: Supplementary file 1 [file Image_1.pdf]

Supplementary Figure S1 Amyloid-ß 2

Supplementary Figure S2 Tau 3

Supplementary Figure S3 HSP70 4

Supplementary Figure S4 HSP90 5

Supplementary Figure S5 HSPB1 6

Supplementary Figure S6 BAG3 7

Supplementary Figure S7 HSC70 8

Supplementary Figure S8 DNAJB1 9

Supplementary Figure S9 AHA1 10

Supplementary Figure S10 CHIP 11

S1 Amyloid-ß peptide

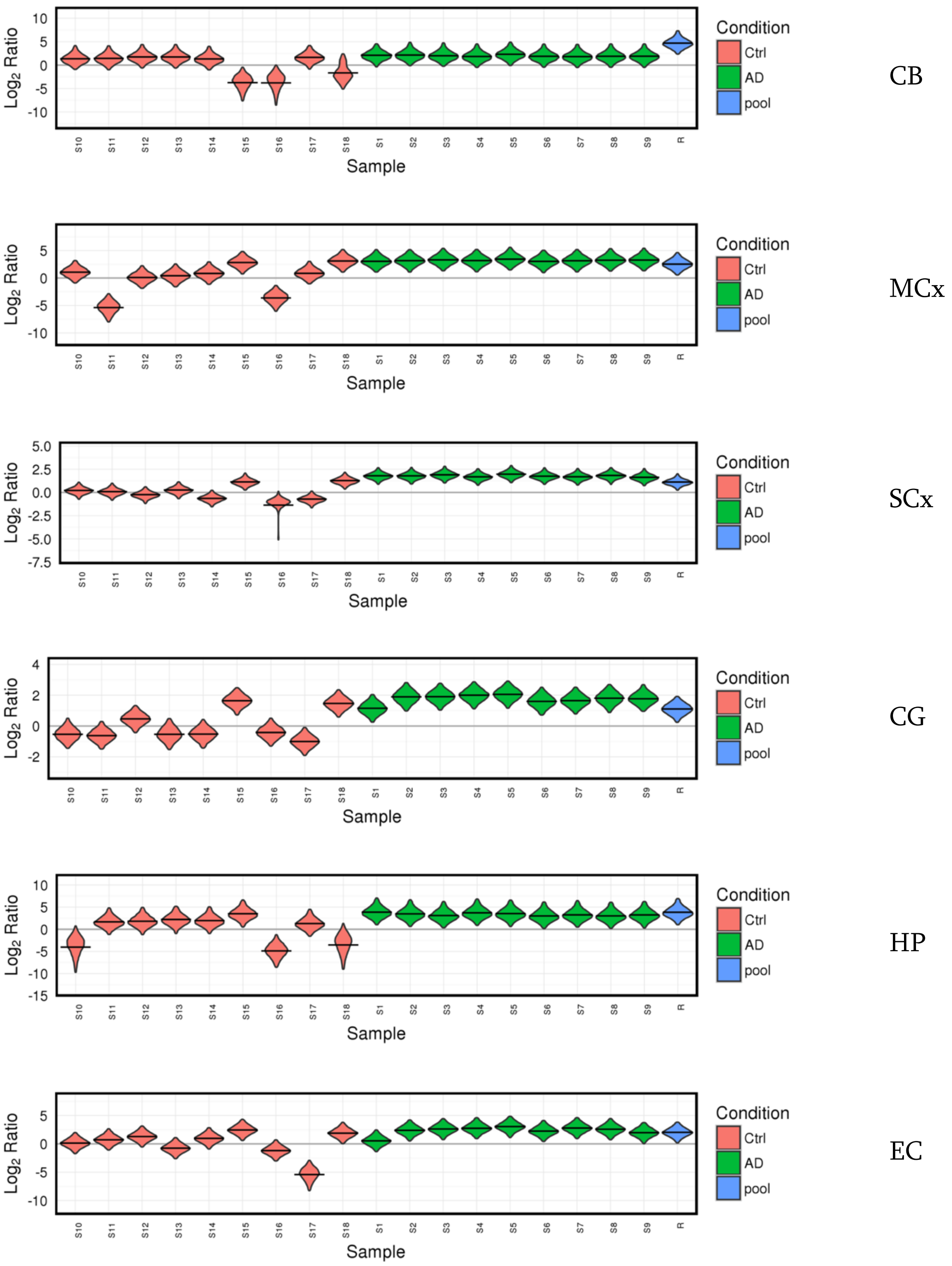

Supplementary Figure S1.
Overview per brain region of Bayesian probability distribution for estimated levels of Amyloid-ß peptide 40/42 with datapoints for every individual analysed sample. Brain regions are indicated in abbreviations on the right side of the graph (CB = cerebellum, MCx = motor cortex, SCx = sensory cortex, CG = cingulate gyrus, HP = hippocampus and EC = entorhinal cortex).

S2 Tau

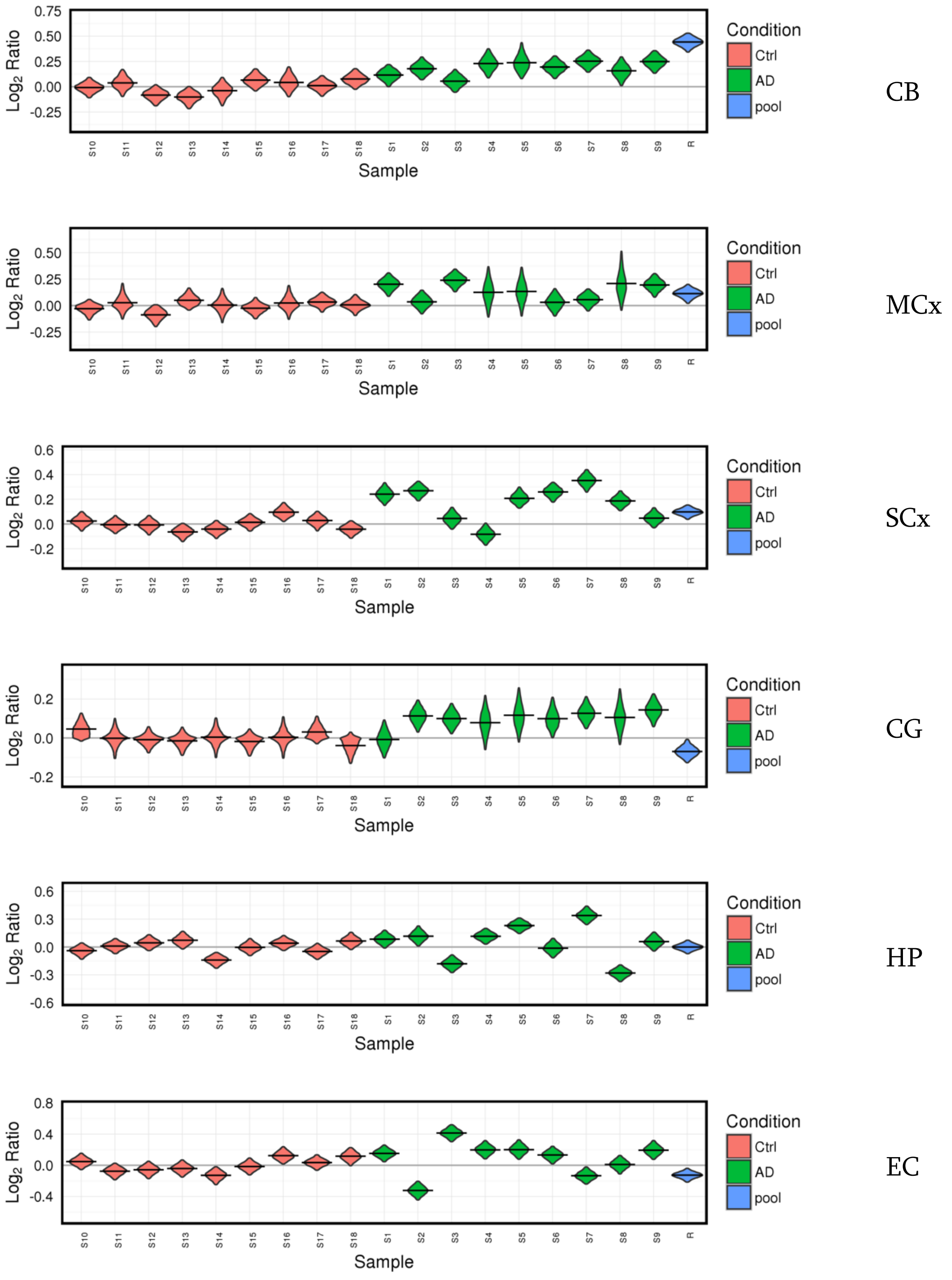

Supplementary Figure S2.
Overview per brain region of Bayesian probability distribution for estimated levels of Tau with datapoints for every individual analysed sample. Brain regions are indicated in abbreviations on the right side of the graph (CB = cerebellum, MCx = motor cortex, SCx = sensory cortex, CG = cingulate gyrus, HP = hippocampus and EC = entorhinal cortex).

S3 HSP70

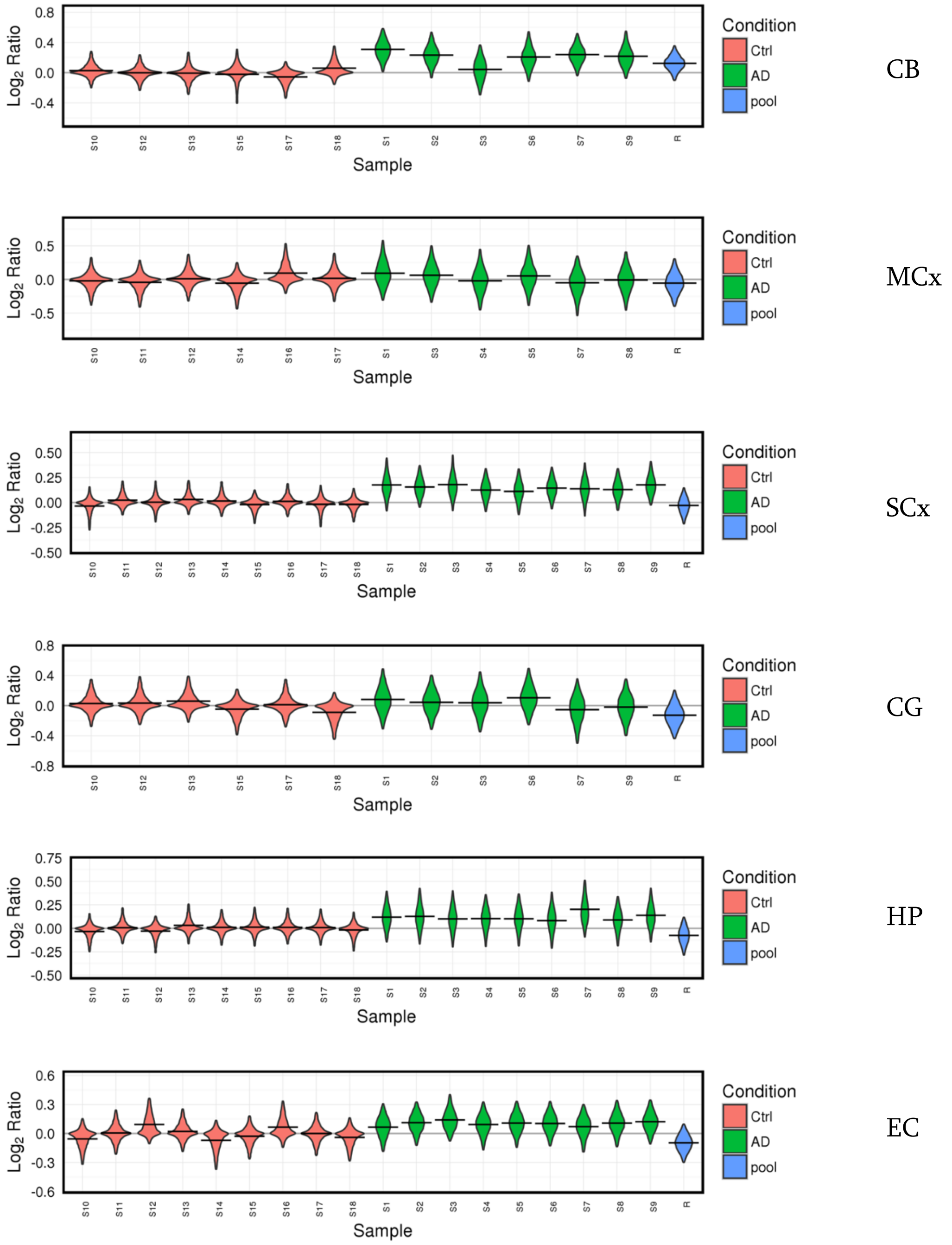

Supplementary Figure S3.
Overview per brain region of Bayesian probability distribution for estimated levels of Hsp70 with datapoints for every individual analysed sample. Brain regions are indicated in abbreviations on the right side of the graph (CB = cerebellum, MCx = motor cortex, SCx = sensory cortex, CG = cingulate gyrus, HP = hippocampus and EC = entorhinal cortex).

S4 HSP90

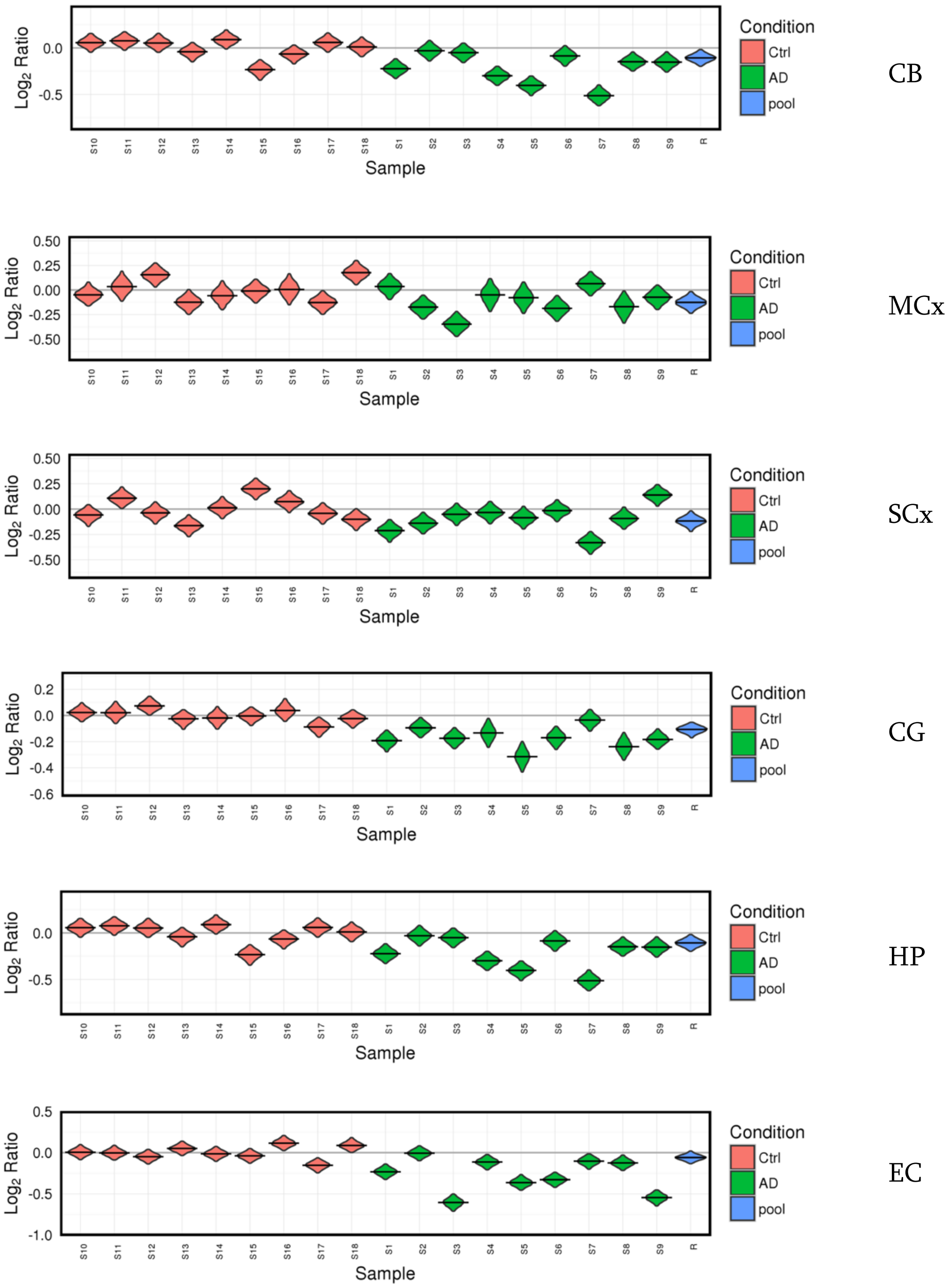

Supplementary Figure S4.
Overview per brain region of Bayesian probability distribution for estimated levels of HSP90 with datapoints for every individual analysed sample. Brain regions are indicated in abbreviations on the right side of the graph (CB = cerebellum, MCx = motor cortex, SCx = sensory cortex, CG = cingulate gyrus, HP = hippocampus and EC = entorhinal cortex).

S5 HSPB1

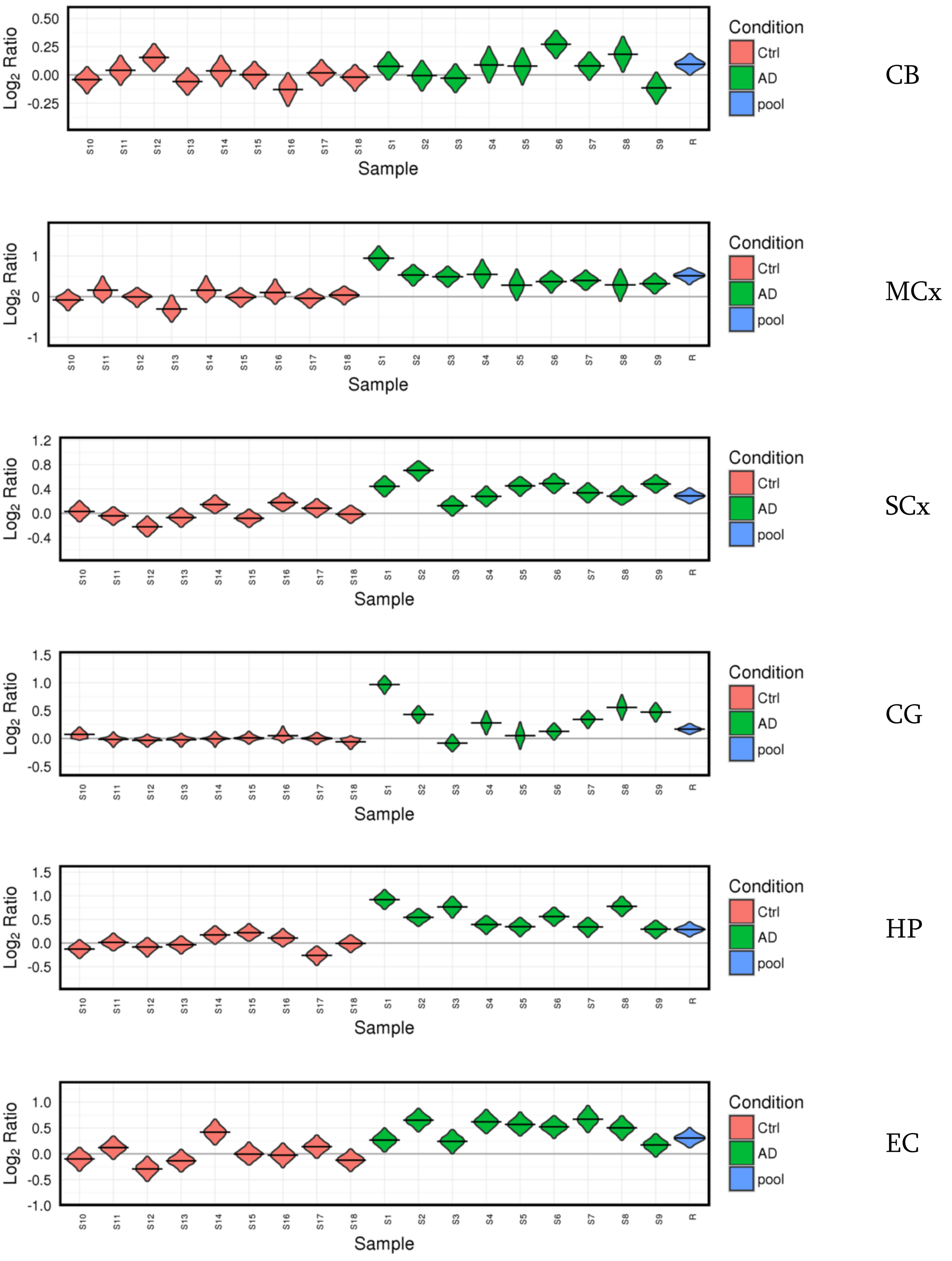

Supplementary Figure S5.
Overview per brain region of Bayesian probability distribution for estimated levels of HSPB1 with datapoints for every individual analysed sample. Brain regions are indicated in abbreviations on the right side of the graph (CB = cerebellum, MCx = motor cortex, SCx = sensory cortex, CG = cingulate gyrus, HP = hippocampus and EC = entorhinal cortex).

S6 BAG3

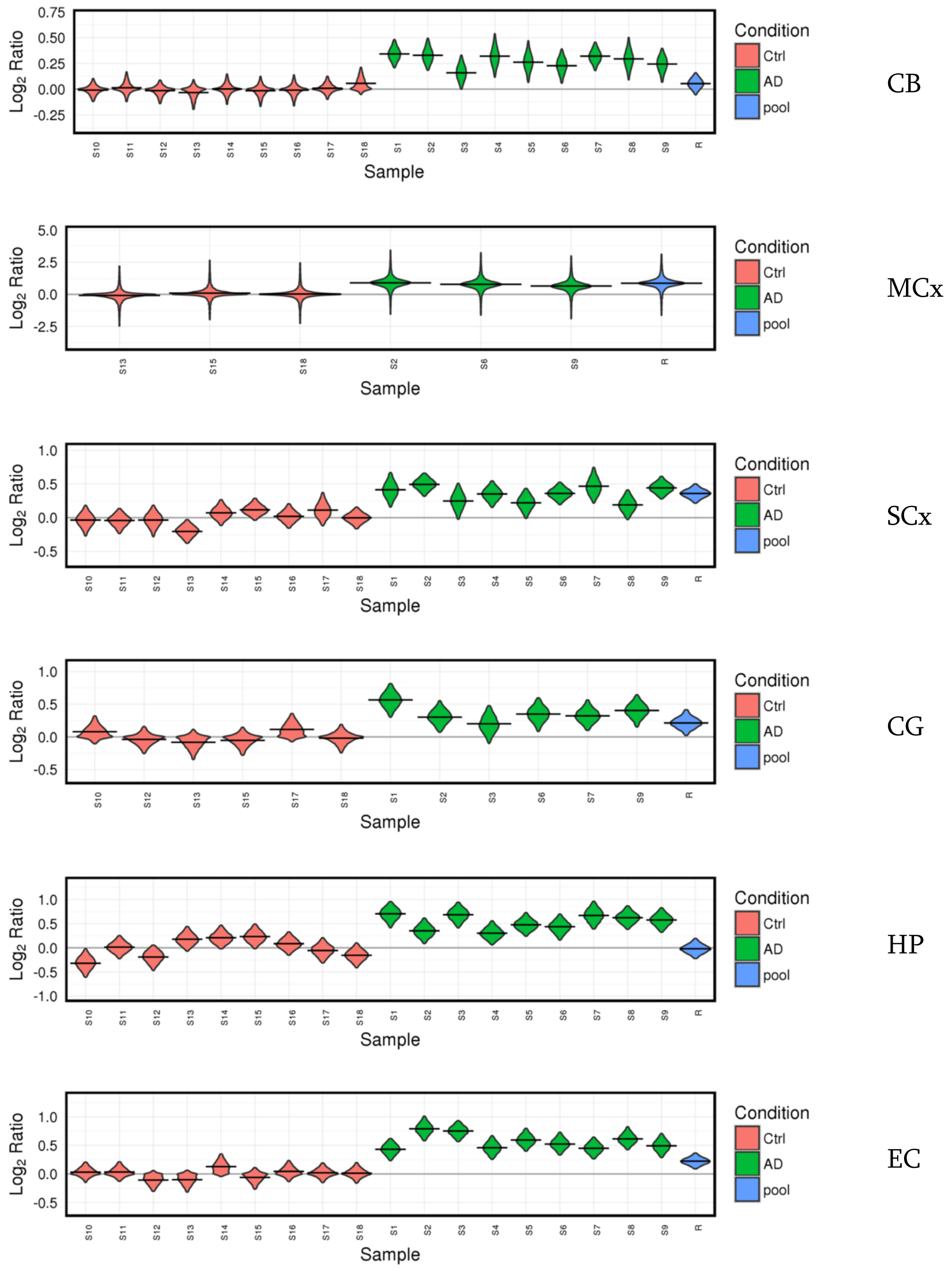

Supplementary Figure S6.
Overview per brain region of Bayesian probability distribution for estimated levels of BAG3 with datapoints for every individual analysed sample. Brain regions are indicated in abbreviations on the right side of the graph (CB = cerebellum, MCx = motor cortex, SCx = sensory cortex, CG = cingulate gyrus, HP = hippocampus and EC = entorhinal cortex).

S7 HSC70

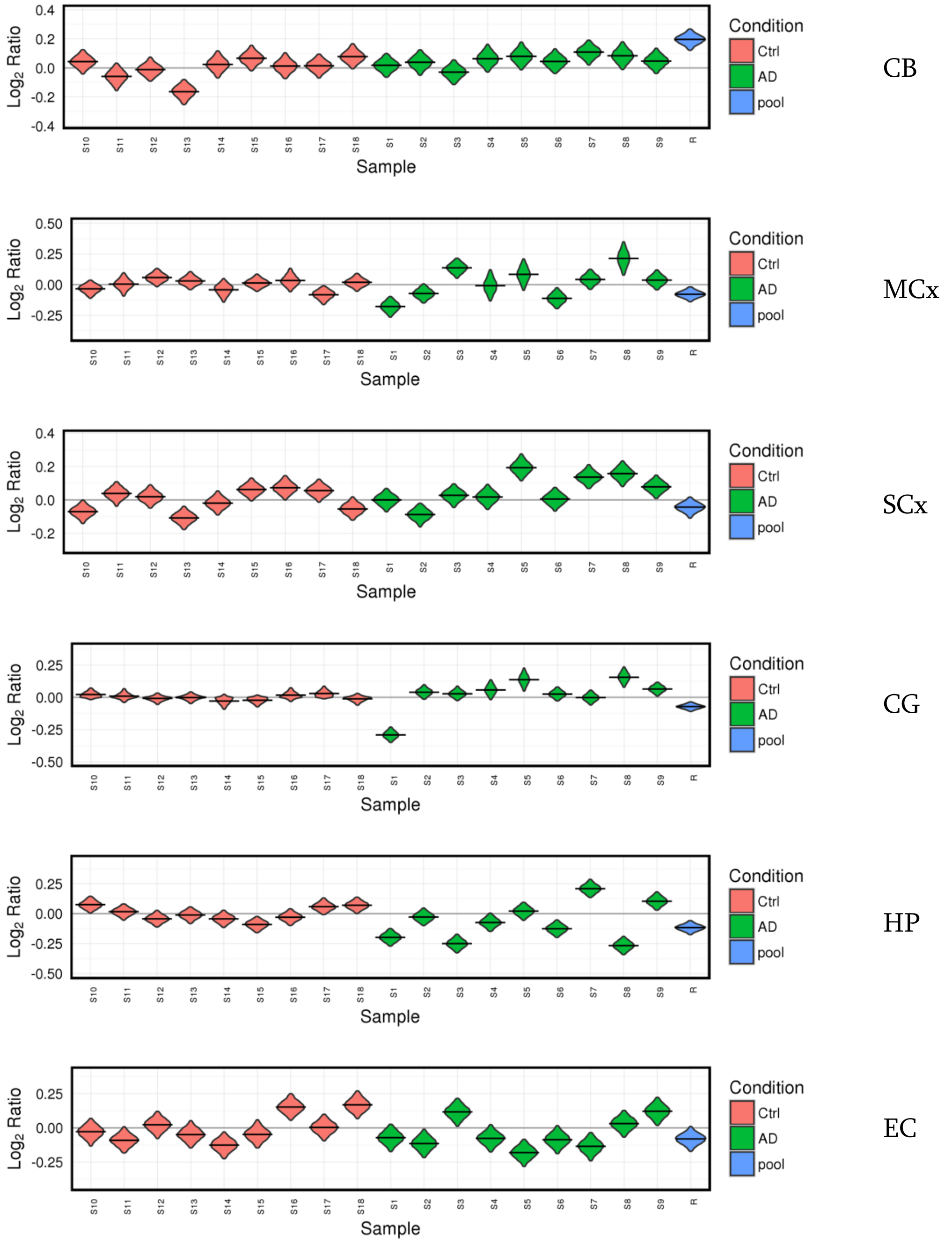

Supplementary Figure S7.
Overview per brain region of Bayesian probability distribution for estimated levels of HSC70 with datapoints for every individual analysed sample. Brain regions are indicated in abbreviations on the right side of the graph (CB = cerebellum, MCx = motor cortex, SCx = sensory cortex, CG = cingulate gyrus, HP = hippocampus and EC = entorhinal cortex).

S8 DNAJB1

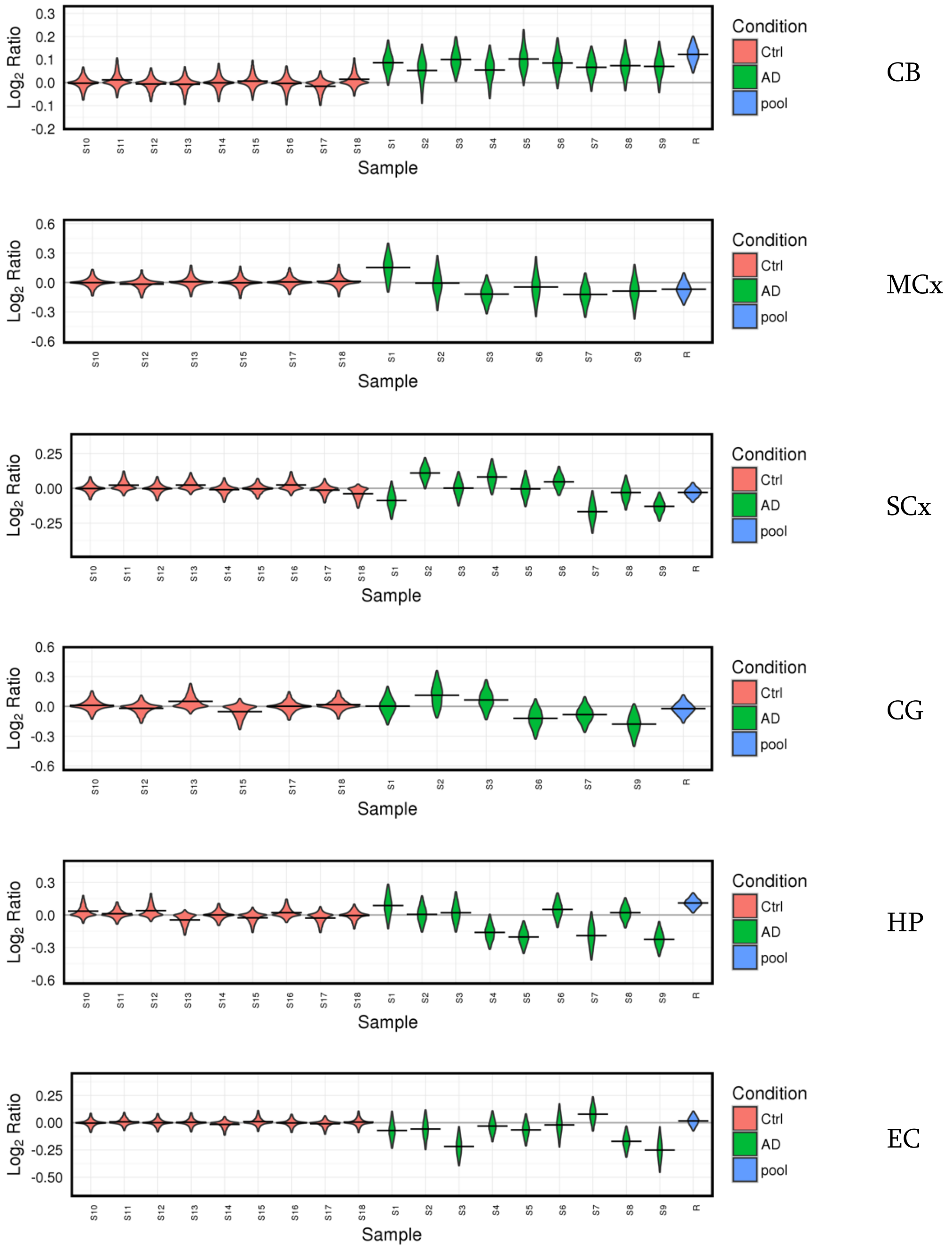

Supplementary Figure S8.
Overview per brain region of Bayesian probability distribution for estimated levels of DNAJB1 with datapoints for every individual analysed sample. Brain regions are indicated in abbreviations on the right side of the graph (CB = cerebellum, MCx = motor cortex, SCx = sensory cortex, CG = cingulate gyrus, HP = hippocampus and EC = entorhinal cortex).

S9 AHA1

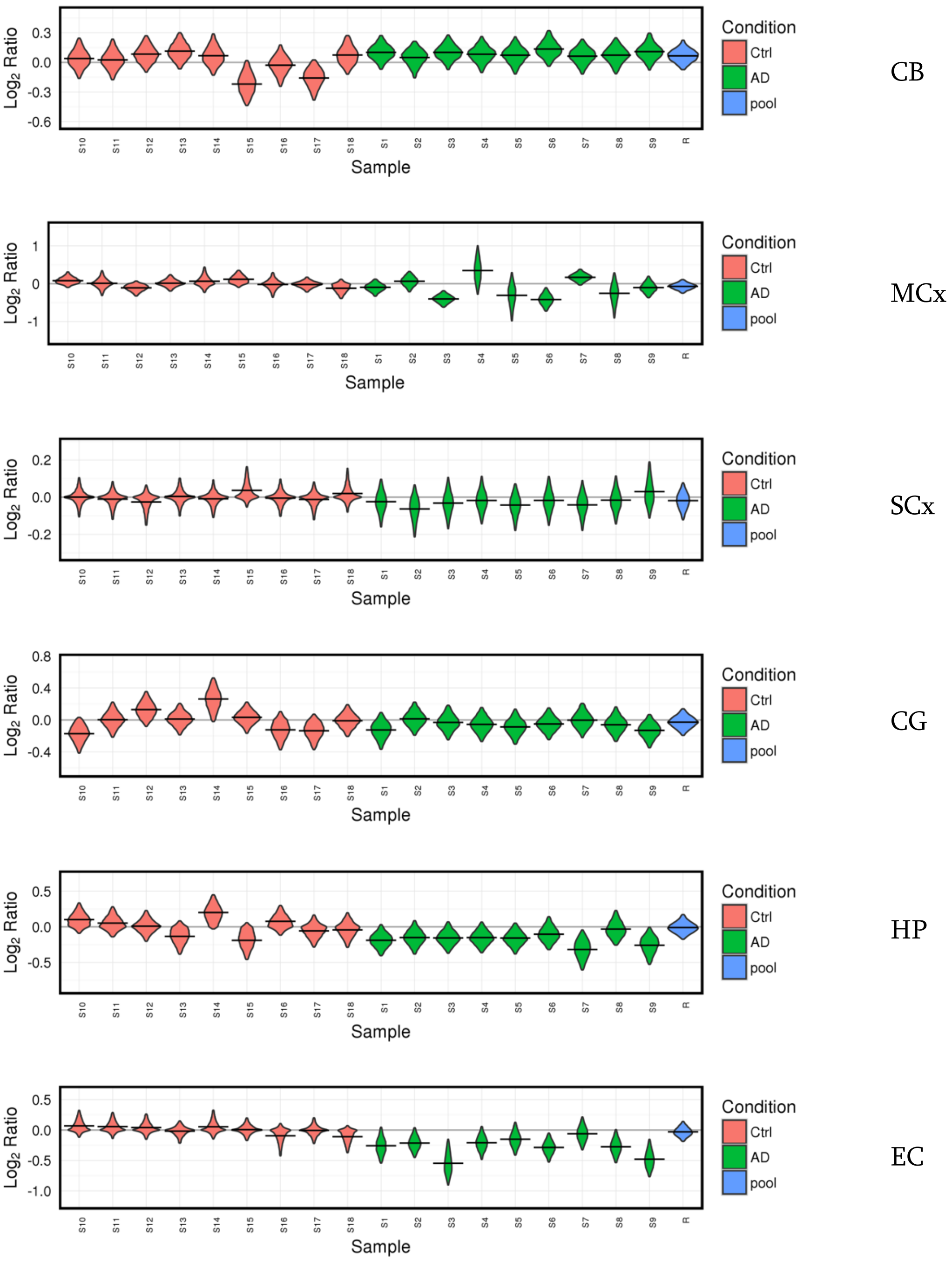

Supplementary Figure S9.
Overview per brain region of Bayesian probability distribution for estimated levels of AHA1 with datapoints for every individual analysed sample. Brain regions are indicated in abbreviations on the right side of the graph (CB = cerebellum, MCx = motor cortex, SCx = sensory cortex, CG = cingulate gyrus, HP = hippocampus and EC = entorhinal cortex).

S10 CHIP

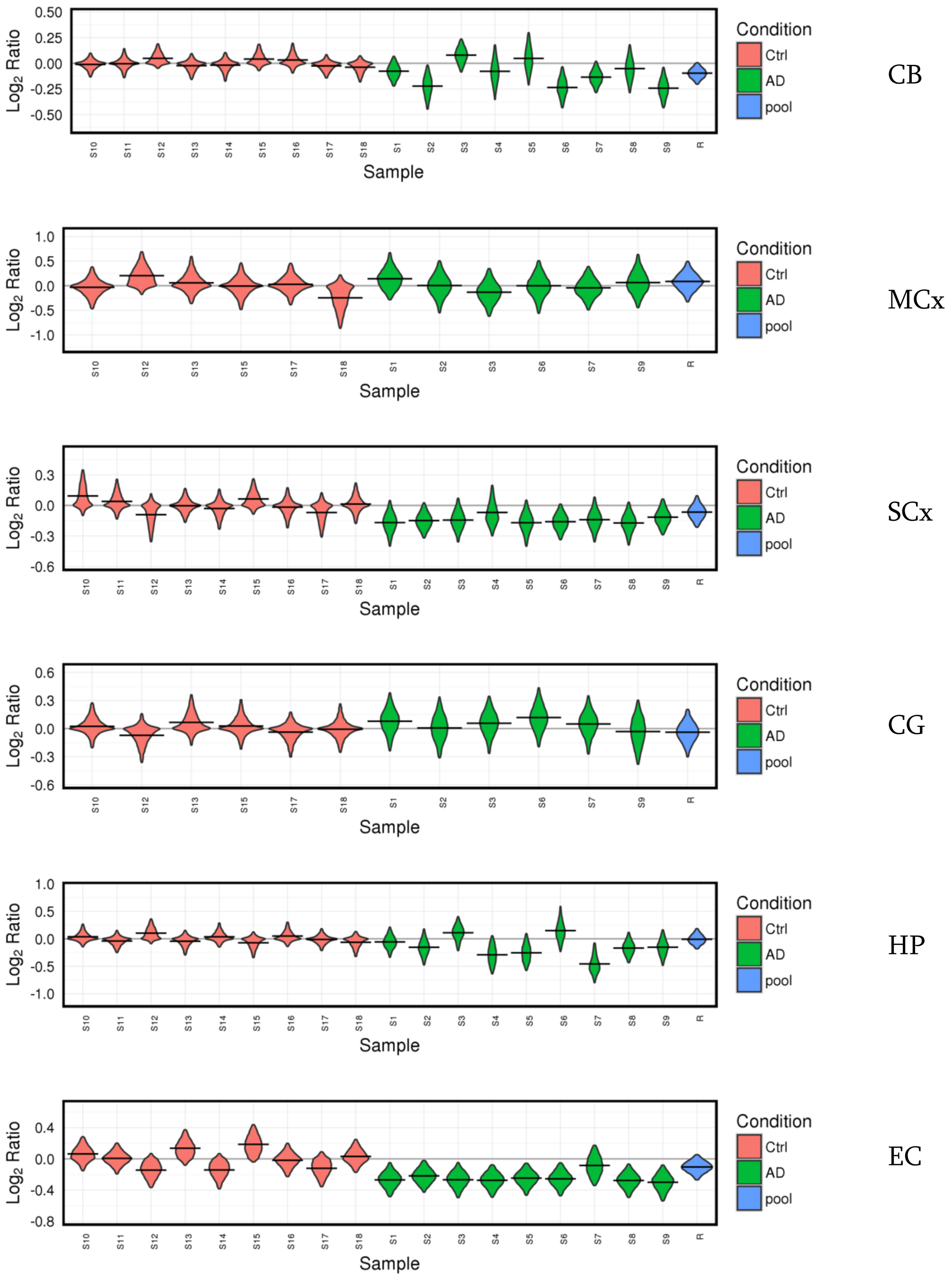

Supplementary Figure S10.
Overview per brain region of Bayesian probability distribution for estimated levels of CHIP with datapoints for every individual analysed sample. Brain regions are indicated in abbreviations on the right side of the graph (CB = cerebellum, MCx = motor cortex, SCx = sensory cortex, CG = cingulate gyrus, HP = hippocampus and EC = entorhinal cortex).
